# Role of essential and trace elements in chronic myeloid leukemia: associations with serum trace element profiles and hematological parameters

**DOI:** 10.3389/fragi.2026.1920709

**Published:** 2026-09-14

**Authors:** Ajay Kumar Yadav, Umesh Choudhary, Anupam Aich, Lalit Prashant Meena, Royana Singh, Shivam Tiwari, Ashish Ashish

**Affiliations:** 1 Department of Anatomy, Institute of Medical Science, Banaras Hindu University, Varanasi, India; 2 Department of General Medicine, Institute of Medical Science, Banaras Hindu University, Varanasi, India; 3 Multidisciplinary Research Unit, ICMR-DHR, Institute of Medical Sciences, Banaras Hindu University, Varanasi, India

**Keywords:** chronic myeloid leukemia, copper, iron, selenium, serum biomarkers, trace element profiling, zinc

## Abstract

**Background:**

Chronic myeloid leukemia is a myeloproliferative malignancy characterized by the Philadelphia chromosome with uncontrolled expansion of myeloid cells. Trace elements are essential for cellular metabolism, DNA repair, antioxidant defence, and immune regulation, and their imbalance may influence leukemogenesis and contribute to oxidative stress, immune dysregulation, and hematological alterations. Profiling trace elements may improve understanding of CML-associated trace element alterations and identify candidate biomarkers for future validation.

**Material and methods:**

A cross-sectional study at Banaras Hindu University, India, included 93 CML patients and 93 age and sex-matched healthy controls from an eastern Indian population. Serum chromium (Cr), copper (Cu), manganese (Mn), lead (Pb), cadmium (Cd), arsenic (As), iron (Fe), selenium (Se), and zinc (Zn) concentrations were measured by atomic absorption spectroscopy. Complete blood counts were performed, and data analysed using descriptive statistics, Mann–Whitney U test, Chi-square/Fisher’s exact tests, and Spearman correlation analysis.

**Results:**

CML patients exhibited significantly elevated serum concentrations of Cu, Mn, Pb, Cd, and As compared with healthy controls, whereas Cr, Fe, Se, and Zn concentrations were significantly higher in healthy controls. Several trace elements showed statistically significant associations with hematological parameters, suggesting complex interactions between elemental imbalance and CML biology.

**Conclusion:**

This study strongly shows dysregulation of essential and toxic trace elements in CML. Imbalance of selenium (Se), zinc (Zn), iron (Fe), manganese (Mn), and copper (Cu) may contribute to oxidative stress, immune dysfunction, and leukemogenesis, and toxic metals may enhance genomic instability. Trace-element profiling, especially selenium-related biomarkers, may serve as candidate biomarkers associated with CML and warrant validation in larger prospective studies.

## Graphical Abstract

**Figure:**
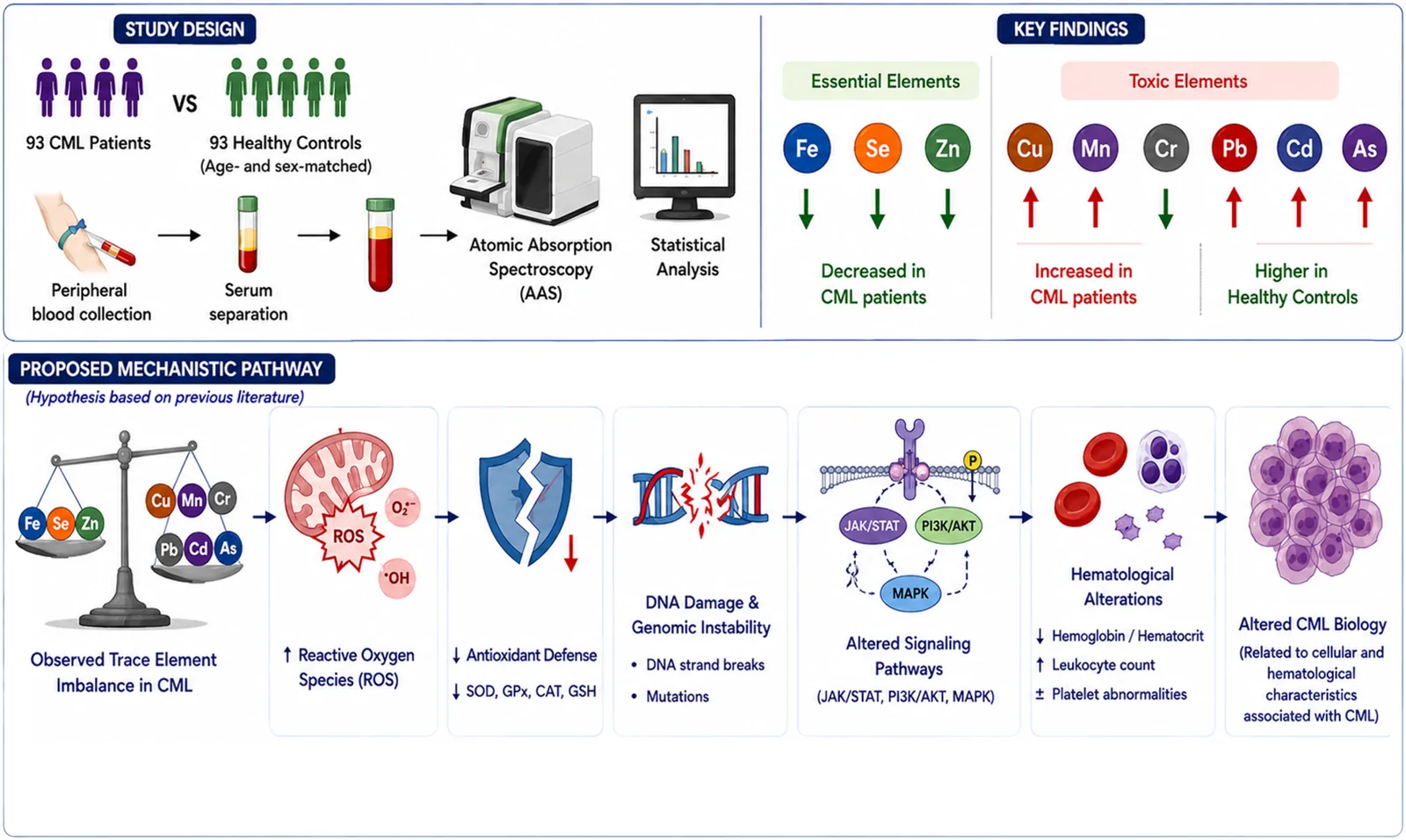
The graphical representation of experimental design.

## Introduction

Chronic myeloid leukemia, a clonal myeloproliferative neoplasm, is characterized by excessive proliferation of total leukocytes counts (TLC) in the peripheral blood characterized by bone marrow, and originating from haematopoietic stem cells in the bone marrow (Mojtahedi et al., 2021). The prevalence of CML in India is approximately 5.47 cases per 100,000 individuals annually. The incidence of CML has increased markedly, with projections indicating a possible stabilization by 2050 (Sarangi et al., 2024). This disease is primarily driven by a reciprocal translocation between chromosomes 9 and 22, resulting in the development of the Philadelphia chromosome and the BCR–ABL1 fusion gene (Benchikh et al., 2022). The BCR-ABL-1 gene encodes an active tyrosine kinase that promotes uninhibited cell division, inhibits apoptosis, and enhances genomic variability, resulting in driving leukemogenesis and disease progression (Amarante-Mendes et al., 2022) CML progresses in mainly three separate phases: chronic phase (CP), accelerated phase (AP), and blast phase (BP). Maximum patients are diagnosed in the chronic phase, which may remain asymptomatic (Senapati et al., 2022). Without effective treatment, CML progresses to the accelerated and blast phases. Tyrosine kinase inhibitors (TKIs), imatinib, dasatinib, and nilotinib, have modernised CML therapy by specifically targeting the BCR–ABL1 kinase (Laganà et al., 2025). TKIs transformed CML into a manageable chronic condition, achieving major molecular or deep molecular responses in patients. The alternative therapeutic options, allogeneic stem cell transplantation, are reserved for patients with TKI failure or intolerance (Alves et al., 2021). Beyond genetic and molecular factors, trace elements play an important role in haematopoiesis, redox balance, and immune regulation, and may influence cancer progression and treatment outcomes (Górska et al., 2024). Trace elements such as iron, zinc, copper, and selenium, serve as cofactors for numerous enzymes involved in DNA repair, antioxidant defense, and cell signaling (Górska et al., 2024). Imbalance of essential micronutrients, or accumulation of toxic metals (cadmium, lead, and arsenic) can promote oxidative stress, DNA damage, and abnormal signal transduction thereby contributing to leukemogenesis and disease advancement (Thakur et al., 2025; Djulejic et al., 2023). TKIs are specifically targeted and inhibit the activity of the BCR-ABL1 tyrosine kinase, suppressing the growth and proliferation of myeloid cells (Bavaro et al., 2019). Previous studies suggest TKIs, imatinib, dasatinib, and nilotinib, significantly improve the prognosis of CML patients, with many achieving long-term remission (Vener et al., 2020). The ongoing researches are focused on finding new therapeutic targets and developing more effective treatments for patients with TKI resistance or intolerance. Despite their recognized importance in various malignancies, the specific patterns and roles of trace element imbalance in CML pathophysiology, prognosis, and therapeutic response remain insufficiently understood (Akanni et al., 2013).

Trace elements are essential micronutrients required for normal cellular function, and maintaining their balance is crucial, as both deficiency and excess can adversely affect health (Espinosa-Salas and Gonzalez-Arias, 2026). These elements play interconnected and complex roles in cellular homeostasis, including DNA synthesis and repair, enzymatic activity, metabolic regulation, nerve conduction, membrane stability, and antioxidant defense (Espinosa-Salas and Gonzalez-Arias, 2026; Górska et al., 2024). Although minerals constitute only a small proportion of the human diet, they are indispensable for sustaining physiological processes (Khaliq et al., 2018). However, evidence regarding their role in the etiology and progression of CML remains limited and inconclusive (Wang et al., 2021).

In this study, we aimed to evaluate the levels of selected trace elements in CML patients and healthy individuals and their association with disease pathogenesis and hematological characteristics. Among essential elements, zinc is a critical cofactor for multiple enzymes implicated in DNA replication, protein synthesis, and cell division (Sharif et al., 2012). It also regulates gene expression through zinc-finger transcription factors and supports immune function, particularly T-cell and natural killer cell activity (Sharif et al., 2012). In CML, increased cellular proliferation and the effects of TKIs may contribute to reduced zinc levels.

Iron is vital for hemoglobin synthesis, oxygen transport, and cellular proliferation (Chen et al., 2019). CML patients are commonly anemic due to chronic inflammation and dysregulation of iron absorption, mostly mediated by increased hepcidin levels, which limit iron absorption and availability (Chen et al., 2019). Copper, an essential element, is involved in angiogenesis, oxidative metabolism, and antioxidant defense (Urso and Maffia, 2016). Elevated copper levels in CML are associated with increased oxidative stress, angiogenesis, and leukemic cell proliferation and may also contribute to resistance to TKI therapy (Labachevski et al., 2023).

Selenium, is known for antioxidant and immunomodulatory properties, plays a protective role against oxidative damage via selenoproteins (glutathione peroxidases) (Alqaisi et al., 2026). Selenium enhances immune surveillance and, in leukemic cells, promotes apoptosis while sparing normal hematopoietic cells, indicating therapeutic potential (Ehudin et al., 2022). Manganese supports enzyme function, antioxidant defense, and cellular signaling, and imbalance may indirectly influence disease progression via altered redox status (Baj et al., 2023).

Toxic trace elements, cadmium, lead, chromium, and arsenic, are associated with carcinogenic effects. Cadmium induces oxidative stress and DNA damage, while lead disrupts hematopoiesis and increases reactive oxygen species production (Balali-Mood et al., 2021). Chromium generates oxidative DNA damage and genomic instability, and arsenic exhibits a dual role, acting as both a therapeutic agent and a carcinogen (Senapati and Sasaki, 2022; Hu et al., 2022). Elevated levels observed in CML patients suggest a potential association with leukemogenesis and altered cellular homeostasis. However, the cross-sectional design does not permit conclusions regarding treatment resistance or disease progression (Lafta et al., 2024). Overall, understanding the role of trace elements may provide insights into the biological characteristics of CML and guide future mechanistic research.

In this study we investigate the serum concentrations of essential (iron, zinc, copper, selenium, manganese) and toxic trace elements (cadmium, lead, arsenic, chromium) in CML patients compared with healthy controls. By elucidating trace element alterations and their correlations with haematological parameters, this study aims to identify candidate biomarkers associated with CML and evaluate their association with hematological parameters.

## Methods and materials

### Study design

This study was conducted in the Department of General Medicine (Hematology), Institute of Medical Sciences, Banaras Hindu University (BHU), Varanasi, including patients who were from eastern Uttar Pradesh and Bihar. After obtaining approval from the Institutional Ethics Committee with ethical approval number (2022/EC/4095), a total of 93 cases and 93 healthy controls were enrolled. Blood samples were collected from participants attending the Hematology Outpatient Department (OPD) and inpatient hematology wards between November 2021 to October 2025. The participants with confirmed diagnosis of CML were included in the study. Blood samples were collected from the OPD, with 6 mL obtained from each participant. Of this, 3 mL was transferred into plain vials and was allowed to clot at room temperature. The serum was separated by centrifugation and aliquoted into pre-labeled cryovials, which were stored at −80 °C until further analysis. Diagnosis of chronic myeloid leukemia (CML) was established by consultant hematologists according to standard institutional diagnostic protocols based on clinical presentation, complete blood count, peripheral blood smear findings, bone marrow examination, and cytogenetic and/or molecular investigations, including detection of the Philadelphia chromosome and/or BCR–ABL1 fusion gene where available. Cytogenetic information presented in this study was obtained retrospectively from hospital records. Because this was a retrospective observational study, complete cytogenetic and molecular diagnostic data were not available for all enrolled participants.

### Inclusion criteria

Adult patients (≥18 years) with a confirmed diagnosis of chronic myeloid leukemia in the chronic phase, based on standard hematological, cytogenetic, and molecular diagnostic criteria, who were willing to participate and provided written informed consent, were included in the study. Healthy controls were age- and sex-matched individuals without any history of hematological malignancy or chronic systemic disease.

### Exclusion criteria

Individuals with acute or chronic inflammatory diseases, chronic liver or kidney disease, autoimmune disorders, other malignancies, pregnancy, recent blood transfusion, or acute infection were excluded. Participants with a history of mineral or vitamin supplementation within the preceding 3 months, occupational exposure to heavy metals, or known environmental exposure to toxic metals, whenever identified during history taking, were also excluded.

### Atomic absorption spectrophotometer (AAS)

Serum samples were aimed at analyzing trace elements (Mn, Fe, Cu, Pb, Zn, As, Cr, Cd, and Se) using AAS in a PerkinElmer PinAAcle 900T Graphite Furnace Atomic Absorption Spectrometer (PerkinElmer Inc., Waltham, MA, United States). Samples have been diluted 1:10 by 0.4% Triton-X 100 with 1% HNO_3_. The concentrations of elements were measured in μg/dL at the following wavelengths: Mn (279.5), Pb (283.3), Cd (228.8), Fe (248.33), Zn (213.86), Cu (324.75), Se (196.03), and Cr (397.87).

### Calibration curve

Calibration of the standardization for Mn, Pb, Cd, Fe, Cu, Zn, Se, Cr, and As was conducted on the concentration of 1 ppm in a 1% HNO_3_ solution. Serial dilutions of standards 1, 2, and 3 were prepared for AAS at 20 ppb (4, 6, and 8 ppb). Zn and Fe standards were primarily set to 1 ppm and then subsequently diluted to 50 ppb. Standards of 1, 2, and 3 were adjusted to concentrations of 10 ppb, 20 ppb, and 30 ppb, correspondingly. The absorbance measurements of both the standards and blanks were plotted versus their concentrations using a 0.997 correlation coefficient (*R* ^2^). Samples were kept until the end of atomic absorption spectroscopy analysis. For the white blood cell (WBC) differential count, an automated hematology analyzer (Sysmex XN-1000, Sysmex Corporation, Kobe, Japan) was used to perform a complete blood count (CBC), including total WBC count and differential leukocyte analysis. Atomic absorption spectroscopy was used to determine important trace element as described by Bernhard (1985).

### Statistical analysis

Descriptive and inferential statistical analysis was performed by using the Statistical Package for the Social Sciences (SPSS) software Ver 26.0 (Chicago, United States). The distribution of continuous variables was assessed using the Shapiro–Wilk test. Because the trace element concentrations were not normally distributed, continuous variables are presented as median (interquartile range) or mean rank, as appropriate. Comparisons between CML patients and healthy controls were performed using the Mann–Whitney U test. Categorical variables were compared using the Chi-square test or Fisher’s exact test, as appropriate. Correlations between trace element concentrations and hematological parameters were evaluated using Spearman’s rank correlation coefficient. A two-sided P value <0.05 was considered statistically significant.

## Results

During the comparison, the demographic analysis divided the participants into two groups, healthy control and CML patients, and found no significant differences in age, gender, geographical location, dietary habits, smoking status, and family income, as shown in Table 1. The only exception was in the educational level: a significantly higher percentage of individuals in the control group had higher secondary or graduate education compared to the case group, and this difference was statistically significant (p = 0.010).

**TABLE 1:** Demographic characteristics of the study participants (N = 186).

Variable	Category	Case group n (%)	Control group n (%)	χ^2^	p
Age category	≤20	8 (8.6)	5 (5.4)	6.87	0.076
	21–40	78 (83.9)	62 (66.7)		
	41–60	2 (2.2)	18 (19.4)		
	61–80	5 (5.4)	8 (8.6)		
Gender	Male	50 (53.8)	36 (38.7)	3.24	0.072
	Female	43 (46.2)	57 (61.3)		
State	U.P.	71 (76.3)	71 (76.3)	0.00	1.00
	Bihar	22 (23.7)	22 (23.7)		
Smoking/Alcohol	None	78 (83.9)	77 (82.8)	7.14	0.128
	Alcoholic	5 (5.4)	2 (2.2)		
	Tobacco	8 (8.6)	6 (6.5)		
	Gul powder	0 (0.0)	5 (5.4)		
	Other	2 (2.2)	3 (3.2)		
Occupation	Hard workers	65 (69.9)	65 (69.9)	0.00	1.00
	Professional	22 (23.7)	22 (23.7)		
	Business/Self-employed	6 (6.5)	6 (6.5)		
Family income	<50,000	80 (86.0)	80 (86.0)	0.00	1.00
	50,000 to 1 lac	8 (8.6)	8 (8.6)		
	>100,000	5 (5.4)	5 (5.4)		
Diet	Vegetarian	36 (38.7)	50 (53.8)	3.33	0.068
	Mixed	57 (61.3)	43 (46.2)		
Education	Primary	22 (23.7)	8 (8.6)	11.34	0.010*
	Upper secondary	35 (37.6)	33 (35.5)		
	Higher secondary	22 (23.7)	32 (34.4)		
	Graduate	14 (15.1)	20 (21.5)		

* Values are presented as frequency (percentage). Percentages are based on valid responses. Group 1 (Case) and Group 2 (control) represent the two study groups. χ2 = Pearson chi-square test. p < 0.05.

Most participants showed positive karyotyping results (44.1%), while 20.4% had negative results; the remaining 35.5% had no available data. The majority were in the chronic disease phase (82.8%), with smaller numbers in the accelerated (12.9%) and blast (4.3%) phases. The prevalence of comorbid conditions was low, with only a few individuals reporting tuberculosis, thyroid disorders, or hypertension (Table 2).

**TABLE 2:** Demographic and clinical characteristics of the sample (N = 93).

Variable	Category	Frequency (n)	Percent (%)
Karyotyping	NA	33	35.5
	Positive	41	44.1
	Negative	19	20.4
Stage of disease	Chronic phase	77	82.8
	Accelerated phase	12	12.9
	Blast phase	4	4.3
Other diseases	None (NA)	87	93.5
	BP	1	1.1
	TB	3	3.2
	TSH	2	2.2

Percentages are based on the total sample (N = 93). BP, blood pressure; TB, tuberculosis; TSH, Thyroid-related disorder.

Analysis of trace-element concentrations revealed significant differences between the two The CML group had significantly higher serum concentrations of copper (Cu), manganese (Mn), lead (Pb), cadmium (Cd), and arsenic (As), whereas the control group exhibited significantly higher concentrations of chromium (Cr), iron (Fe), selenium (Se), and zinc (Zn) (Table 3) Haematological parameters showed significant links with haemoglobin and PCV positively correlated, while both negatively associated with TLC and RDW-CV. Additionally, iron was positively associated with cadmium, and manganese was inversely associated with iron.

**TABLE 3:** Comparison of trace element concentrations between groups (n = 186).

Variable	Case (n = 93) mean rank	Control (n = 93) mean rank	U	Z	p (2-tailed)
Cr (µg/L)	52.94	134.06	552.50	−10.28	<0.001
Fe (µg/L)	47.84	139.16	78.00	−11.57	<0.001
Cu (µg/L)	131.74	55.26	768.00	−9.69	<0.001
Se (µg/L)	55.06	131.94	750.00	−9.74	<0.001
Zn (µg/L)	49.95	137.05	274.00	−11.04	<0.001
Mn (µg/L)	134.68	52.32	495.00	−10.43	<0.001
Pb (µg/L)	124.48	62.52	1,443.00	−7.85	<0.001
Cd (µg/L)	122.45	64.55	1,632.50	−7.34	<0.001
As (µg/L)	127.61	59.39	1,152.00	−8.65	<0.001

Mann–Whitney U tests were conducted to compare the distribution of trace element concentrations between two groups. Mean ranks are reported for each group. All comparisons were statistically significant at *p* < 0.001.

Results from the Mann-Whitney U tests represent significant variances in trace element concentrations between the two groups. The CML group showed significantly higher concentrations of manganese (Mn), copper (Cu), lead (Pb), cadmium (Cd), and arsenic (As) (*p < 0.001*), while control had significantly higher concentrations of iron (Fe; *p < 0.001*), selenium (Se; *p < 0.001*), chromium (Cr; *p < 0.001*) and zinc (Zn; *p < 0.001*) as shown in Table 3.

The correlation matrix indicated several significant associations among trace elements. Chromium (Cr) showed positive correlations with cadmium (Cd; r = 0.492, p < 0.001) and manganese (Mn; r = 0.568, p < 0.001). Iron (Fe) showed a significant negative correlation with Mn (r = −0.692, *p = 0.000*) and a negative correlation with Cd (r = −0.353, *p = 0.000*). Copper (Cu) showed a negative correlation with selenium (Se; r = −0.579, *p = 0.000*) and a positive correlation with Mn (r = 0.526, *p = 0.000*). Other associations were not statistically significant, as shown in Table 4.

**TABLE 4:** Spearman’s rank-order correlations among trace elements (N = 93).

Variable		Cr (µg/L)	Fe (µg/L)	Cu (µg/L)	Se (µg/L)	Zn (µg/L)	Mn (µg/L)	Pb (µg/L)	Cd (µg/L)	As (µg/L)
Cr (µg/L)	Correlation coefficient	1.000	−0.613^**^	0.558^**^	−0.580^**^	−0.652^**^	0.568^**^	0.479^**^	0.492^**^	0.518^**^
	Sig. (2-Tailed)	​	0.000	0.000	0.000	0.000	0.000	0.000	0.000	0.000
	N	186	186	186	186	186	186	186	186	186
Fe (µg/L)	Correlation coefficient	−0.613^**^	1.000	−0.641^**^	0.637^**^	0.680^**^	−0.692^**^	−0.530^**^	−0.353^**^	−0.571^**^
	Sig. (2-Tailed)	0.000	​	0.000	0.000	0.000	0.000	0.000	0.000	0.000
	N	186	186	186	186	186	186	186	186	186
Cu (µg/L)	Correlation coefficient	0.558^**^	−0.641^**^	1.000	−0.579^**^	−0.569^**^	0.526^**^	0.439^**^	0.351^**^	0.373^**^
	Sig. (2-Tailed)	0.000	0.000	​	0.000	0.000	0.000	0.000	0.000	0.000
	N	186	186	186	186	186	186	186	186	186
Se (µg/L)	Correlation coefficient	−0.580^**^	0.637^**^	−0.579^**^	1.000	0.611^**^	−0.581^**^	−0.474^**^	−0.404^**^	−0.427^**^
	Sig. (2-Tailed)	0.000	0.000	0.000	​	0.000	0.000	0.000	0.000	0.000
	N	186	186	186	186	186	186	186	186	186
Zn (µg/L)	Correlation coefficient	−0.652^**^	0.680^**^	−0.569^**^	0.611^**^	1.000	−0.592^**^	−0.502^**^	−0.507^**^	−0.539^**^
	Sig. (2-Tailed)	0.000	0.000	0.000	0.000	​	0.000	0.000	0.000	0.000
	N	186	186	186	186	186	186	186	186	186
Mn (µg/L)	Correlation coefficient	0.568^**^	−0.692^**^	0.526^**^	−0.581^**^	−0.592^**^	1.000	0.393^**^	0.402^**^	0.468^**^
	Sig. (2-Tailed)	0.000	0.000	0.000	0.000	0.000	​	0.000	0.000	0.000
	N	186	186	186	186	186	186	186	186	186
Lead (µg/L)	Correlation coefficient	0.479^**^	−0.530^**^	0.439^**^	−0.474^**^	−0.502^**^	0.393^**^	1.000	0.394^**^	0.263^**^
	Sig. (2-Tailed)	0.000	0.000	0.000	0.000	0.000	0.000	​	0.000	0.000
	N	186	186	186	186	186	186	186	186	186
Cd (µg/L)	Correlation coefficient	0.492^**^	−0.353^**^	0.351^**^	−0.404^**^	−0.507^**^	0.402^**^	0.394^**^	1.000	0.305^**^
	Sig. (2-Tailed)	0.000	0.000	0.000	0.000	0.000	0.000	0.000	​	0.000
	N	186	186	186	186	186	186	186	186	186
As (µg/L)	Correlation coefficient	0.518^**^	−0.571^**^	0.373^**^	−0.427^**^	−0.539^**^	0.468^**^	0.263^**^	0.305^**^	1.000
	Sig. (2-Tailed)	0.000	0.000	0.000	0.000	0.000	0.000	0.000	0.000	​
	N	186	186	186	186	186	186	186	186	186

Values represent Spearman's rho correlation coefficients. * Indicates *p* < 0.05, and ** indicates *p* < 0.01. Coefficients above the diagonal are omitted for clarity.

Haematological parameters revealed, hemoglobin/PCV was positively correlated with HCT/PCV (*r*_*s*_ = 0.623, *p = 0.000*) and negatively correlated with TLC (*r*_*s*_ = −0.447, *p = 0.000*) and RDW-CV (*r*_*s*_ = −0.572, *p = 0.000*) as shown in Table 5. TLC showed strong positive associations with MCHC (*r*_*s*_ = 0.150, *p = 0.041*) and platelet count (*r*_*s*_ = 0.346, *p = 0.000*), and a negative correlation with lymphocytes (*r*_*s*_ = −0.566, *p = 0.000*). Among trace elements, Fe was negatively associated with Cd (*r*_*s*_ = −0.353, *p = 0.000*), whereas Mn was inversely correlated with Fe (*r*_*s*_ = −0.692, *p = 0.000*).

**TABLE 5:** Spearman’s rank correlations among haematological and trace element variables (N = 93).

Variable		HB/PCV (g/dL)	TLC (thou/mm3)	Neutrophils (%)	Lymphocytes (%)	HCT/PCV (%)	Mcv (fL)	Mch (pg)	MCHC (g/dL)	RDW-CV%)
HB/PCV (g/dL)	Correlation coefficient	1.000	−0.447^**^	0.134	0.463^**^	0.623^**^	0.246^**^	0.207^**^	0.222^**^	−0.572^**^
	Sig. (2-Tailed)		0.000	0.068	0.000	0.000	0.001	0.005	0.002	0.000
	N	186	186	186	186	186	186	186	186	186
TLC (thou/mm3)	Correlation coefficient	−0.447^**^	1.000	0.103	−0.566^**^	−0.414^**^	−0.269^**^	0.016	0.150^*^	0.383^**^
	Sig. (2-Tailed)	0.000		0.161	0.000	0.000	0.000	0.824	0.041	0.000
	N	186	186	186	186	186	186	186	186	186
Neutrophils (%)	Correlation coefficient	0.134	0.103	1.000	0.124	0.134	0.136	−0.041	0.040	−0.116
	Sig. (2-Tailed)	0.068	0.161		0.093	0.069	0.064	0.576	0.583	0.113
	N	186	186	186	186	186	186	186	186	186
Lymphocytes (%)	Correlation coefficient	0.463^**^	−0.566^**^	0.124	1.000	0.517^**^	0.185^*^	−0.011	−0.023	−0.413^**^
	Sig. (2-Tailed)	0.000	0.000	0.093		0.000	0.012	0.880	0.758	0.000
	N	186	186	186	186	186	186	186	186	186
HCT/PCV (%)	Correlation coefficient	0.623^**^	−0.414^**^	0.134	0.517^**^	1.000	0.223^**^	0.110	0.105	−0.481^**^
	Sig. (2-Tailed)	0.000	0.000	0.069	0.000		0.002	0.136	0.153	0.000
	N	186	186	186	186	186	186	186	186	186
MCV (fL)	Correlation coefficient	0.246^**^	−0.269^**^	0.136	0.185^*^	0.223^**^	1.000	0.310^**^	0.015	−0.106
	Sig. (2-Tailed)	0.001	0.000	0.064	0.012	0.002		0.000	0.837	0.148
	N	186	186	186	186	186	186	186	186	186
MCH (pg)	Correlation coefficient	0.207^**^	0.016	−0.041	−0.011	0.110	0.310^**^	1.000	0.279^**^	−0.120
	Sig. (2-Tailed)	0.005	0.824	0.576	0.880	0.136	0.000		0.000	0.102
	N	186	186	186	186	186	186	186	186	186
MCHC (g/dL)	Correlation coefficient	0.222^**^	0.150^*^	0.040	−0.023	0.105	0.015	0.279^**^	1.000	−0.264^**^
	Sig. (2-Tailed)	0.002	0.041	0.583	0.758	0.153	0.837	0.000		0.000
	N	186	186	186	186	186	186	186	186	186
RDW-CV (%)	Correlation coefficient	−0.572^**^	0.383^**^	−0.116	−0.413^**^	−0.481^**^	−0.106	−0.120	−0.264^**^	1.000
	Sig. (2-Tailed)	0.000	0.000	0.113	0.000	0.000	0.148	0.102	0.000	
	N	186	186	186	186	186	186	186	186	186
RDW-SD (fL)	Correlation coefficient	−0.469^**^	0.330^**^	−0.050	−0.416^**^	−0.421^**^	−0.099	−0.105	−0.155^*^	0.602^**^
	Sig. (2-Tailed)	0.000	0.000	0.497	0.000	0.000	0.177	0.155	0.035	0.000
	N	186	186	186	186	186	186	186	186	186
Platelets (thou/mm3)	Correlation coefficient	−0.188^*^	0.346^**^	0.068	−0.274^**^	−0.095	−0.166^*^	−0.013	0.249^**^	0.260^**^
	Sig. (2-Tailed)	0.010	0.000	0.360	0.000	0.195	0.023	0.863	0.001	0.000
	N	186	186	186	186	186	186	186	186	186
Cr (Microgram/L)	Correlation coefficient	−0.543^**^	0.364^**^	−0.129	−0.418^**^	−0.533^**^	−0.157^*^	−0.134	−0.216^**^	0.516^**^
	Sig. (2-Tailed)	0.000	0.000	0.080	0.000	0.000	0.032	0.069	0.003	0.000
	N	186	186	186	186	186	186	186	186	186
Fe case (Microgram/L))	Correlation coefficient	0.548^**^	−0.411^**^	0.165^*^	0.454^**^	0.543^**^	0.163^*^	0.058	0.203^**^	−0.607^**^
	Sig. (2-Tailed)	0.000	0.000	0.025	0.000	0.000	0.026	0.433	0.006	0.000
	N	186	186	186	186	186	186	186	186	186
Cu(Microgram/L)	Correlation coefficient	−0.544^**^	0.375^**^	−0.051	−0.346^**^	−0.432^**^	−0.152^*^	−0.063	−0.141	0.510^**^
	Sig. (2-Tailed)	0.000	0.000	0.489	0.000	0.000	0.039	0.395	0.055	0.000
	N	186	186	186	186	186	186	186	186	186
Se (selenium) (Microgram/L)	Correlation coefficient	0.433^**^	−0.308^**^	0.117	0.385^**^	0.465^**^	0.046	0.075	0.200^**^	−0.447^**^
	Sig. (2-Tailed)	0.000	0.000	0.110	0.000	0.000	0.534	0.311	0.006	0.000
	N	186	186	186	186	186	186	186	186	186
Zn(Microgram/L))	Correlation coefficient	0.589^**^	−0.401^**^	0.177^*^	0.454^**^	0.510^**^	0.176^*^	0.079	0.249^**^	−0.594^**^
	Sig. (2-Tailed)	0.000	0.000	0.016	0.000	0.000	0.016	0.284	0.001	0.000
	N	186	186	186	186	186	186	186	186	186
Mn (Microgram/L)	Correlation coefficient	−0.411^**^	0.380^**^	−0.101	−0.382^**^	−0.463^**^	−0.153^*^	−0.197^**^	−0.221^**^	0.576^**^
	Sig. (2-Tailed)	0.000	0.000	0.168	0.000	0.000	0.037	0.007	0.002	0.000
	N	186	186	186	186	186	186	186	186	186
Lead (Microgram/L))	Correlation coefficient	−0.356^**^	0.241^**^	−0.130	−0.335^**^	−0.391^**^	−0.070	−0.047	−0.125	0.421^**^
	Sig. (2-Tailed)	0.000	0.001	0.077	0.000	0.000	0.344	0.525	0.088	0.000
	N	186	186	186	186	186	186	186	186	186
Cd(Microgram/L))	Correlation coefficient	−0.344^**^	0.247^**^	−0.032	−0.294^**^	−0.308^**^	−0.163^*^	−0.195^**^	−0.199^**^	0.400^**^
	Sig. (2-Tailed)	0.000	0.001	0.660	0.000	0.000	0.026	0.008	0.006	0.000
	N	186	186	186	186	186	186	186	186	186
As (Microgram/L))	Correlation coefficient	−0.487^**^	0.240^**^	−0.097	−0.300^**^	−0.467^**^	−0.034	−0.097	−0.214^**^	0.474^**^
	Sig. (2-Tailed)	0.000	0.001	0.188	0.000	0.000	0.641	0.187	0.003	0.000
	N	186	186	186	186	186	186	186	186	186

RDW-SD (fL)	Platelets (thou/mm^3^)	Cr (µg/L)	Fe (µg/L)	Cu (µg/L)	Se (µg/L)	Zn (µg/L)	Mn (µg/L)	Pb (µg/L)	Cd (µg/L)	As (µg/L)
−0.469^**^	−0.188^*^	−0.543^**^	0.548^**^	−0.544^**^	0.433^**^	0.589^**^	−0.411^**^	−0.356^**^	−0.344^**^	−0.487^**^
0.000	0.010	0.000	0.000	0.000	0.000	0.000	0.000	0.000	0.000	0.000
186	186	186	186	186	186	186	186	186	186	186
0.330^**^	0.346^**^	0.364^**^	−0.411^**^	0.375^**^	−0.308^**^	−0.401^**^	0.380^**^	0.241^**^	0.247^**^	0.240^**^
0.000	0.000	0.000	0.000	0.000	0.000	0.000	0.000	0.001	0.001	0.001
186	186	186	186	186	186	186	186	186	186	186
−0.050	0.068	−0.129	0.165^*^	−0.051	0.117	0.177^*^	−0.101	−0.130	−0.032	−0.097
0.497	0.360	0.080	0.025	0.489	0.110	0.016	0.168	0.077	0.660	0.188
186	186	186	186	186	186	186	186	186	186	186
−0.416^**^	−0.274^**^	−0.418^**^	0.454^**^	−0.346^**^	0.385^**^	0.454^**^	−0.382^**^	−0.335^**^	−0.294^**^	−0.300^**^
0.000	0.000	0.000	0.000	0.000	0.000	0.000	0.000	0.000	0.000	0.000
186	186	186	186	186	186	186	186	186	186	186
−0.421^**^	−0.095	−0.533^**^	0.543^**^	−0.432^**^	0.465^**^	0.510^**^	−0.463^**^	−0.391^**^	−0.308^**^	−0.467^**^
0.000	0.195	0.000	0.000	0.000	0.000	0.000	0.000	0.000	0.000	0.000
186	186	186	186	186	186	186	186	186	186	186
−0.099	−0.166^*^	−0.157^*^	0.163^*^	−0.152^*^	0.046	0.176^*^	−0.153^*^	−0.070	−0.163^*^	−0.034
0.177	0.023	0.032	0.026	0.039	0.534	0.016	0.037	0.344	0.026	0.641
186	186	186	186	186	186	186	186	186	186	186
−0.105	−0.013	−0.134	0.058	−0.063	0.075	0.079	−0.197^**^	−0.047	−0.195^**^	−0.097
0.155	0.863	0.069	0.433	0.395	0.311	0.284	0.007	0.525	0.008	0.187
186	186	186	186	186	186	186	186	186	186	186
−0.155^*^	0.249^**^	−0.216^**^	0.203^**^	−0.141	0.200^**^	0.249^**^	−0.221^**^	−0.125	−0.199^**^	−0.214^**^
0.035	0.001	0.003	0.006	0.055	0.006	0.001	0.002	0.088	0.006	0.003
186	186	186	186	186	186	186	186	186	186	186
0.602^**^	0.260^**^	0.516^**^	−0.607^**^	0.510^**^	−0.447^**^	−0.594^**^	0.576^**^	0.421^**^	0.400^**^	0.474^**^
0.000	0.000	0.000	0.000	0.000	0.000	0.000	0.000	0.000	0.000	0.000
186	186	186	186	186	186	186	186	186	186	186
1.000	0.174^*^	0.466^**^	−0.567^**^	0.479^**^	−0.478^**^	−0.551^**^	0.487^**^	0.369^**^	0.339^**^	0.395^**^
	0.018	0.000	0.000	0.000	0.000	0.000	0.000	0.000	0.000	0.000
186	186	186	186	186	186	186	186	186	186	186
0.174^*^	1.000	0.046	−0.120	0.147^*^	−0.077	−0.053	0.023	0.052	−0.048	−0.018
0.018		0.530	0.102	0.045	0.297	0.474	0.758	0.485	0.518	0.811
186	186	186	186	186	186	186	186	186	186	186
0.466^**^	0.046	1.000	−0.613^**^	0.558^**^	−0.580^**^	−0.652^**^	0.568^**^	0.479^**^	0.492^**^	0.518^**^
0.000	0.530		0.000	0.000	0.000	0.000	0.000	0.000	0.000	0.000
186	186	186	186	186	186	186	186	186	186	186
−0.567^**^	−0.120	−0.613^**^	1.000	−0.641^**^	0.637^**^	0.680^**^	−0.692^**^	−0.530^**^	−0.353^**^	−0.571^**^
0.000	0.102	0.000		0.000	0.000	0.000	0.000	0.000	0.000	0.000
186	186	186	186	186	186	186	186	186	186	186
0.479^**^	0.147^*^	0.558^**^	−0.641^**^	1.000	−0.579^**^	−0.569^**^	0.526^**^	0.439^**^	0.351^**^	0.373^**^
0.000	0.045	0.000	0.000		0.000	0.000	0.000	0.000	0.000	0.000
186	186	186	186	186	186	186	186	186	186	186
−0.478^**^	−0.077	−0.580^**^	0.637^**^	−0.579^**^	1.000	0.611^**^	−0.581^**^	−0.474^**^	−0.404^**^	−0.427^**^
0.000	0.297	0.000	0.000	0.000		0.000	0.000	0.000	0.000	0.000
186	186	186	186	186	186	186	186	186	186	186
−0.551^**^	−0.053	−0.652^**^	0.680^**^	−0.569^**^	0.611^**^	1.000	−0.592^**^	−0.502^**^	−0.507^**^	−0.539^**^
0.000	0.474	0.000	0.000	0.000	0.000		0.000	0.000	0.000	0.000
186	186	186	186	186	186	186	186	186	186	186
0.487^**^	0.023	0.568^**^	−0.692^**^	0.526^**^	−0.581^**^	−0.592^**^	1.000	0.393^**^	0.402^**^	0.468^**^
0.000	0.758	0.000	0.000	0.000	0.000	0.000		0.000	0.000	0.000
186	186	186	186	186	186	186	186	186	186	186
0.369^**^	0.052	0.479^**^	−0.530^**^	0.439^**^	−0.474^**^	−0.502^**^	0.393^**^	1.000	0.394^**^	0.263^**^
0.000	0.485	0.000	0.000	0.000	0.000	0.000	0.000		0.000	0.000
186	186	186	186	186	186	186	186	186	186	186
0.339^**^	−0.048	0.492^**^	−0.353^**^	0.351^**^	−0.404^**^	−0.507^**^	0.402^**^	0.394^**^	1.000	0.305^**^
0.000	0.518	0.000	0.000	0.000	0.000	0.000	0.000	0.000		0.000
186	186	186	186	186	186	186	186	186	186	186
0.395^**^	−0.018	0.518^**^	−0.571^**^	0.373^**^	−0.427^**^	−0.539^**^	0.468^**^	0.263^**^	0.305^**^	1.000
0.000	0.811	0.000	0.000	0.000	0.000	0.000	0.000	0.000	0.000	
186	186	186	186	186	186	186	186	186	186	186

Values are Spearman correlation coefficients. p < .05 is marked with *; p < 0.01 is marked with **.

Interpretation and Correlation with Trace Elements in CML: In CML, abnormal RTK signalling, mainly via BCR-ABL, leads to increased proliferation and survival of leukemic cells. Previous studies suggest that alterations in Fe, Cu, Zn, Mn, and Se may influence oxidative stress and cellular signaling pathways implicated in CML biology. Previous studies have reported that elevated Cu and Mn levels may contribute to increased oxidative stress and altered signaling activity, strengthening oncogenic signals, while Reduced Zn and Se concentrations may impair antioxidant defenses and thereby influence oxidative stress-related signaling pathways. Altered trace element homeostasis may be associated with oxidative stress and signaling pathways that have been implicated in CML pathophysiology. However, pathway activation, oxidative stress markers, and treatment response were not directly investigated in the present study. Therefore, this figure represents a literature-based conceptual mechanism and should not be interpreted as experimental evidence generated from this study shown in Figure 1 **.**

**FIGURE 1:**
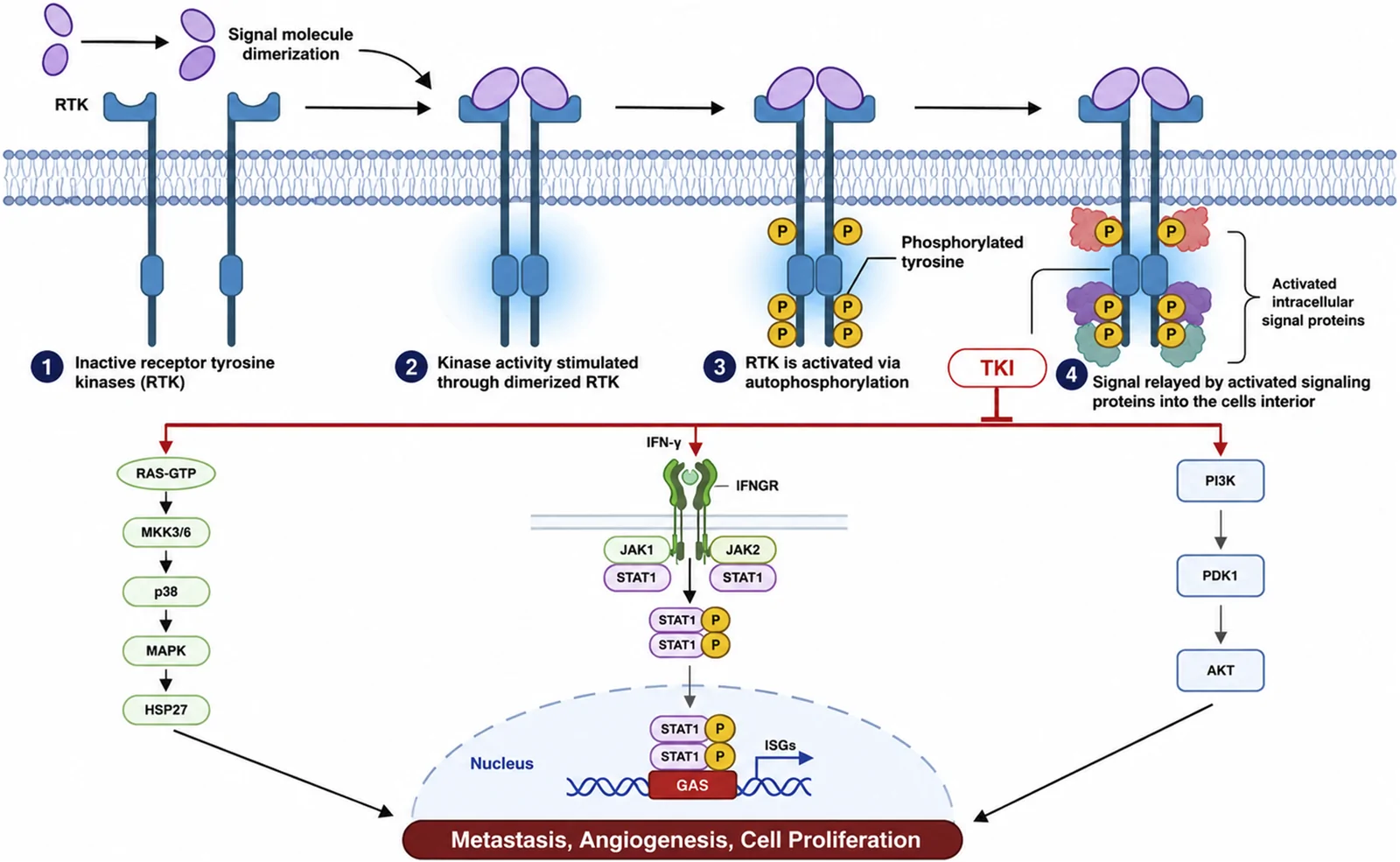
Conceptual diagram illustrating BCR–ABL1 signaling in chronic myeloid leukemia. The schematic shows constitutive activation of the BCR–ABL1 tyrosine kinase and its downstream RAS/MAPK, PI3K/AKT, and JAK/STAT signaling pathways, and indicates that tyrosine kinase inhibitors suppress aberrant signaling. This figure is a literature-based conceptual illustration and does not represent experimental findings from the present study.

## Discussion

In the present study, we observed significant alterations in serum concentrations of essential trace elements and toxic heavy metals in patients with chronic myeloid leukemia compared with healthy controls. Selenium, iron, and zinc concentrations were significantly lower, whereas copper, manganese, cadmium, lead, and arsenic concentrations were significantly higher in CML patients. Furthermore, several trace elements showed significant associations with hematological parameters, suggesting that trace element imbalance is associated with the biological characteristics of CML. These findings are discussed below in the context of previous studies (Lafta et al., 2024). Selenium, iron, copper, Manganese, Lead, Arsenic, Chromium, Cadmium, and zinc are essential micronutrients involved in different physiological, antioxidant defense, DNA synthesis, immune function, and cellular proliferation processes (Lungu et al., 2022; Jomova et al., 2025; Górska et al., 2024). Variations of the levels of trace elements have been linked to carcinogenesis and tumor progression (Mulware, 2012). Several studies have already reported lower selenium levels in cancer patients compared to healthy individuals, directing their important role in cancer development and treatment. A low selenium level has been linked with an increased risk of certain cancers, including colorectal, lung, prostate, and breast cancer, and high levels of selenium have been observed in some malignancies, highlighting the complexity of its involvement in cancer biology (Radomska et al., 2021; Ying and Zhang, 2019). Iron is an important trace element implicated in cancer progression, involved in DNA synthesis and cell proliferation, and an essential nutrient for both normal and cancerous cells (Khan et al., 2020). Tumors often exhibit increased iron uptake to support their rapid growth (Forciniti et al., 2020). Monitoring serum iron levels can aid in the detection of iron dysregulation, providing insights into cancer presence and progression (Lelièvre et al., 2020).

Copper acts as a cofactor for different enzymes involved in angiogenesis and metastasis that are implicated in cancer development. Many studies have suggested that different malignancies, including breast, lung, and colorectal cancer, exhibit high copper levels (Ge et al., 2022). Evaluation of serum copper concentration may suggest valuable information for tumor angiogenesis and potential therapeutic targets (Gao and Zhang, 2023).

Zinc, an essential element for different enzymatic and transcription factors, is also vital for DNA repair, cell growth, and apoptosis. Imbalanced zinc levels reported in different cancers, including prostate, breast, and lung cancer (Costa et al., 2023). Deficiency of zinc can impair immune function and increase cancer susceptibility; zinc accumulation has been associated with tumor growth and metastasis (Wang et al., 2020). Several studies have demonstrated selenium supplementation can significantly suppress the progression of leukemia in murine CML models (Gandhi et al., 2014).

Correlation results suggest different biochemical profiles that can be associated with physiological and environmental factors (Miranda-Galvis et al., 2025). Correlation analyses revealed meaningful relationships between trace elements and hematological parameters; for example, chromium was positively correlated with cadmium and manganese. On the other hand, iron exhibited a negative correlation with cadmium and manganese. These findings show the novelty of our study, as no prior research has reported a positive correlation between serum chromium, cadmium, and manganese levels specifically in CML patients (Esen et al., 2021).

The significant associations between trace elements and hematological parameters constitute one of the principal findings of this study. Hemoglobin and hematocrit showed positive correlations with iron, selenium, and zinc, whereas inverse correlations were observed with chromium, copper, manganese, cadmium, lead, and arsenic. Similarly, total leukocyte count demonstrated positive associations with chromium, copper, manganese, cadmium, lead, and arsenic but negative associations with iron, selenium, and zinc. These findings suggest that disturbances in trace element homeostasis are closely associated with hematological abnormalities in CML and may reflect alterations in erythropoiesis, oxidative stress, and inflammatory responses. However, these associations should be interpreted as observational and do not imply causality.

Manganese and copper are imperative for enzymatic and cellular actions and may contribute to oxidative stress, genomic instability, and altered signaling if present in excess (Jomova et al., 2025). Cadmium, lead, and arsenic are toxic metals, act as potential co-carcinogens, inducing oxidative and genotoxic stress, disturbing DNA repair mechanisms, and promoting irregular haematopoietic regulation (Liu et al., 2026; Sharma et al., 2019) (Figure 1). Direct causal relationships between these metals and CML remain poorly understood; elevated levels observed in CML patients suggest a potential association with leukemogenesis and disease-related biochemical alterations (Balali-Mood et al., 2021). The imbalance between essential and toxic metals may worsen oxidative stress and inflammatory responses, and aggravating disease pathology. Monitoring trace element profiles may improve understanding of the biochemical alterations associated with CML. The potential clinical relevance of correcting trace element imbalance or reducing environmental exposure requires validation in future prospective studies. Further mechanistic and prospective studies are required to clarify the biological significance of these associations and determine their potential clinical relevance. These findings indicate complex interactions between trace elements and hematological indices that warrant further investigation.

## Limitations

This study has several limitations. First, the cross-sectional design precludes conclusions regarding causality, disease progression, prognosis, treatment response, or the predictive value of serum trace elements. Second, the study was conducted at a single center with a relatively modest sample size, which may limit the generalizability of the findings. Third, although demographic characteristics were comparable between groups, residual confounding from unmeasured factors such as detailed dietary intake, environmental exposure, and other lifestyle variables cannot be completely excluded. Fourth, mechanistic pathways linking trace element imbalance to CML were not directly investigated; therefore, the proposed biological mechanisms are based on previous literature rather than experimental evidence from the present study. Finally, multivariable analyses, receiver operating characteristic (ROC) analysis, and correction for multiple comparisons were not performed, and future studies incorporating these approaches are warranted to further validate the clinical significance of the observed associations. Additionally, detailed information regarding tyrosine kinase inhibitor (TKI) treatment status, duration of therapy, treatment response, and molecular remission was not available for all participants because of the retrospective nature of the study. Consequently, the potential influence of treatment on serum trace element concentrations could not be evaluated. Future prospective longitudinal studies incorporating treatment-related variables are warranted to better define these associations.

## Conclusion

This study demonstrated significant alterations in serum concentrations of essential trace elements and toxic heavy metals in patients with chronic myeloid leukemia (CML) compared with healthy controls. The observed differences and their associations with hematological parameters suggest that trace element imbalance is associated with the biological characteristics of CML. Essential trace elements, including selenium, iron, copper, zinc, and manganese, are involved in antioxidant defense, DNA synthesis, immune regulation, and cellular proliferation, whereas elevated levels of toxic metals such as cadmium, lead, and arsenic have been associated with oxidative stress and genomic instability. The findings provide additional evidence supporting the potential involvement of trace element dysregulation in CML pathophysiology.

Although serum trace element profiling may represent a promising source of candidate biomarkers associated with CML, the cross-sectional nature of this study does not permit conclusions regarding causality, disease progression, prognosis, treatment response, or predictive biomarker performance. Furthermore, the clinical utility of correcting trace element imbalances or reducing environmental exposure to toxic metals remains to be established. Therefore, multicenter prospective longitudinal studies with larger sample sizes and mechanistic investigations are warranted to validate these findings, clarify the biological role of trace element alterations, and determine their potential clinical relevance in CML.

## Data Availability

The raw data supporting the conclusions of this article will be made available by the authors, without undue reservation.

## References

[B1] Akanni E. O. Onuegbu A. J. Adebayo T. O. Eegunranti B. A. Oduola T. (2013). Assessment of some selected trace metals in chronic myeloid leukemia patients in a tertiary health facility in south west Nigeria. Asian J. Med. Sci. 5 (4), 71–75. 10.19026/ajms.5.5487

[B2] Alqaisi O. Subih M. Joseph K. Al-Ghabeesh S. Qirim W. Sijarina L. (2026). Selenium as a complementary therapeutic agent in leukemia: a systematic review of preclinical and clinical evidence. Cancer Adv. 9, e26008. 10.53388/2026926008

[B3] Alves R. Gonçalves A. C. Rutella S. Almeida A. M. Rivas J. D. L. Trougakos I. P. (2021). Resistance to tyrosine kinase inhibitors in chronic myeloid leukemia—from molecular mechanisms to clinical relevance. Cancers 13 (19), 4820. 10.3390/CANCERS13194820 34638304 PMC8508378

[B4] Amarante-Mendes G. P. Rana A. Datoguia T. S. Hamerschlak N. Brumatti G. (2022). BCR-ABL1 tyrosine kinase complex signaling transduction: challenges to overcome resistance in chronic myeloid leukemia. Pharmaceutics 14 (1), 215. 10.3390/PHARMACEUTICS14010215 PMC8780254 35057108

[B5] Baj J. Flieger W. Barbachowska A. Kowalska B. Flieger M. Forma A. (2023). Consequences of disturbing manganese homeostasis. Int. J. Mol. Sci. 24 (19), 14959. 10.3390/IJMS241914959 37834407 PMC10573482

[B6] Balali-Mood M. Naseri K. Tahergorabi Z. Khazdair M. R. Sadeghi M. (2021). Toxic mechanisms of five heavy metals: Mercury, lead, chromium, cadmium, and arsenic. Front. Pharmacol. 12, 643972. 10.3389/FPHAR.2021.643972/FULL PMC8078867 33927623

[B7] Bavaro L. Martelli M. Cavo M. Soverini S. (2019). Mechanisms of disease progression and resistance to tyrosine kinase inhibitor therapy in chronic myeloid leukemia: an update. Int. J. Mol. Sci. 20 (24), 6141. 10.3390/IJMS20246141 PMC6940932 31817512

[B8] Benchikh S. Bousfiha A. El Hamouchi A. Soro S. G. C. Malki A. Nassereddine S. (2022). Chronic myeloid leukemia: cytogenetics and molecular biology’s part in the comprehension and management of the pathology and treatment evolution. Egypt. J. Med. Hum. Genet. 23, 29. 10.1186/s43042-022-00248-2

[B9] Chen Y. Fan Z. Yang Y. Gu C. (2019). Iron metabolism and its contribution to cancer (review). Int. J. Oncol. 54 (4), 1143–1154. 10.3892/IJO.2019.4720/HTML 30968149

[B10] Costa M. I. Sarmento-Ribeiro A. B. Gonçalves A. C. (2023). Zinc: from biological functions to therapeutic potential. Int. J. Mol. Sci. 24 (5), 4822. 10.3390/IJMS24054822 36902254 PMC10003636

[B11] Djulejic V. Petrovic B. Jevtic J. Vujacic M. Clarke B. L. Cirovic A. (2023). The role of cadmium in the pathogenesis of myeloid leukemia in individuals with anemia, deficiencies in vitamin D, zinc, and low calcium dietary intake. J. Trace Elem. Med. Biol. 79, 127263. 10.1016/J.JTEMB.2023.127263 37499549

[B12] Ehudin M. A. Golla U. Trivedi D. Potlakayala S. D. Rudrabhatla S. V. Desai D. (2022). Therapeutic benefits of selenium in hematological malignancies. Int. J. Mol. Sci. 23 (14), 7972. 10.3390/IJMS23147972 35887320 PMC9323677

[B13] Esen R. Yuzuncu V. Yetişgin F. Bilici M. (2021). Evaluation of serum levels of trace elements in myeloproliferative neoplasms: a case-control study. East J. Med. 26 (2), 344–350. 10.5505/ejm.2021.58751

[B14] Espinosa-Salas S. Gonzalez-Arias M. (2026). “Nutrition: micronutrient intake, imbalances, and interventions,” in Statpearls. (Treasure Island, FL: StatPearls Publishing). Available online at: https://www.ncbi.nlm.nih.gov/books/NBK597352/ (Accessed September 21, 2023). 37983324

[B15] Forciniti S. Greco L. Grizzi F. Malesci A. Laghi L. (2020). Iron metabolism in cancer progression. Int. J. Mol. Sci. 21 (6), 2257. 10.3390/IJMS21062257 PMC7139548 32214052

[B16] Gandhi U. H. Kaushal N. Hegde S. Finch E. R. Kudva A. K. Kennett M. J. (2014). Selenium suppresses leukemia through the action of endogenous eicosanoids. Cancer Res. 74 (14), 3890–3901. 10.1158/0008-5472 24872387 PMC4102641

[B17] Gao L. Zhang A. (2023). Copper-instigated modulatory cell mortality mechanisms and progress in oncological treatment investigations. Front. Immunol. 14, 1236063. 10.3389/FIMMU.2023.1236063/FULL PMC10433393 37600774

[B18] Ge E. J. Bush A. I. Casini A. Cobine P. A. Cross J. R. DeNicola G. M. (2022). Connecting copper and cancer: from transition metal signalling to metalloplasia. Nat. Rev. Cancer 22 (2), 102–113. 10.1038/s41568-021-00417-2 34764459 PMC8810673

[B19] Górska A. Markiewicz-Gospodarek A. Trubalski M. Żerebiec M. Poleszak J. Markiewicz R. (2024). Assessment of the impact of trace essential metals on cancer development. Int. J. Mol. Sci. 25 (13), 6842. 10.3390/IJMS25136842 38999951 PMC11241304

[B20] Hu J. Hu B. Deng L. Cheng L. Fan Q. Lu C. (2022). Arsenic sulfide inhibits the progression of gastric cancer through regulating the circRNA_ASAP2/Wnt/ß-catenin pathway. Anticancer Drugs 33 (1), E711–E719. 10.1097/CAD.0000000000001246 34486534 PMC8670347

[B21] Jomova K. Alomar S. Y. Valko R. Nepovimova E. Kuca K. Valko M. (2025). The role of redox-active iron, copper, manganese, and redox-inactive zinc in toxicity, oxidative stress, and human diseases. EXCLI J. 24, 880–954. 10.17179/EXCLI2025-8449 PMC12419454 40933952

[B22] Khaliq H. Juming Z. Ke-Mei P. (2018). The physiological role of boron on health. Biol. Trace Elem. Res. 186 (1), 31–51. 10.1007/S12011-018-1284-3/TABLES/4 29546541

[B23] Khan A. Singh P. Srivastava A. (2020). Iron: key player in cancer and cell cycle? J. Trace Elem. Med. Biol. 62, 126582. 10.1016/J.JTEMB.2020.126582 32673942

[B24] Labachevski B. Popova Labacevska M. Panovska Stavridis I. Trajkova S. Pivkova Veljanovska A. Stojanoski Z. (2023). Assessment and corelation of oxidative stress between healthy adults and adult patients with chronic myeloid leukemia treated with imatinib. J. Morphol. Sci. 6 (1), 51–61. 10.55302/JMS2361051L

[B25] Lafta M. H. Afra A. Patra I. Jalil A. T. Mohammadi M. J. Baqir Al-Dhalimy A. M. (2024). Toxic effects due to exposure heavy metals and increased health risk assessment (leukemia). Rev. Environ. Health 39 (2), 351–362. 10.1515/REVEH-2022-0227/XML 36574711

[B26] Laganà A. Scalzulli E. Bisegna M. L. Ielo C. Martelli M. Breccia M. (2025). Understanding and overcoming resistance to tyrosine kinase inhibitors (TKIs) in chronic myeloid leukemia (CML). Expert Rev. Hematol. 18 (1), 65–79. 10.1080/17474086.2024.2440776 39647915

[B27] Lelièvre P. Sancey L. Coll J. L. Deniaud A. Busser B. (2020). Iron dysregulation in human cancer: altered metabolism, biomarkers for diagnosis, prognosis, monitoring and rationale for therapy. Cancers 12 (12), 3524. 10.3390/CANCERS12123524 33255972 PMC7761132

[B28] Liu X. Han Z. Han K. Pang Y. Zhao X. Wang Y. (2026). Oxidative stress, DNA damage, DNA repair inhibition, and apoptosis induced by lead and cadmium combined exposure in TK6 cells. Toxics 14 (4), 341. 10.3390/TOXICS14040341/S1 PMC13120129 42043168

[B29] Lungu I. I. Babarus I. Oniciuc L. Stefanache A. (2022). A review of essential microelements in the immune system. Int. J. Immunol. 10 (1), 1–4. 10.11648/j.iji.20221001.11

[B30] Miranda-Galvis M. Tjioe K. C. Sharara M. Maloney M. Keruakous A. R. Jillella A. (2025). Estimating the burden of chronic stress through allostatic load in patients with chronic myeloid leukemia. Leukemia 39 (11), 2735–2744. 10.1038/s41375-025-02763-8 40935825

[B31] Mojtahedi H. Yazdanpanah N. Rezaei N. (2021). Chronic myeloid leukemia stem cells: targeting therapeutic implications. Stem Cell Res. Ther. 12 (1), 603. 10.1186/S13287-021-02659-1 34922630 PMC8684082

[B32] Mulware S. J. (2012). Trace elements and carcinogenicity: a subject in review. 3 Biotech. 3 (2), 85–96. 10.1007/S13205-012-0072-6 28324563 PMC3597132

[B33] Radomska D. Czarnomysy R. Radomski D. Bielawska A. Bielawski K. (2021). Selenium as a bioactive micronutrient in the human diet and its cancer chemopreventive activity. Nutrients 13 (5), 1649. 10.3390/NU13051649 34068374 PMC8153312

[B34] Sarangi S. S. Yadav R. Singh R. (2024). Patient perspectives on living with chronic myeloid leukemia in India: quality of life, psychosocial support, and stigma. Int. J. Mol. Immuno Oncol. 9, 71–76. 10.25259/IJMIO_17_2024

[B35] Senapati J. Sasaki K. (2022). Chromosomal instability in chronic myeloid leukemia: mechanistic insights and effects. Cancers 14 (10), 2533. 10.3390/CANCERS14102533 35626137 PMC9140097

[B36] Senapati J. Jabbour E. Kantarjian H. Short N. J. (2022). Pathogenesis and management of accelerated and blast phases of chronic myeloid leukemia. Leukemia 37 (1), 5–17. 10.1038/s41375-022-01736-5 36309558

[B37] Sharif R. Thomas P. Zalewski P. Fenech M. (2012). The role of zinc in genomic stability. Mutat. Res. Fundam. Mol. Mech. Mutagen. 733 (1–2), 111–121. 10.1016/J.MRFMMM.2011.08.009 21939673

[B38] Sharma A. Kumar M. Kaur S. Nagpal A. K. (2019). Evaluation of environmental contaminants and natural products: a human health perspective. Eval. Environ. Contam. Nat. Prod. A Hum. Health Perspective 9, 1–296. 10.2174/97898114109631190101

[B39] Thakur R. Singh S. Chauhan A. Kumar P. Saini R. V. Saini A. K. (2025). Lead and arsenic toxicity: emerging mechanisms, immunotoxic effects, and future research perspectives. BioMetals 38 (5), 1421–1445. 10.1007/S10534-025-00717-2 40614026

[B40] Urso E. Maffia M. (2016). Behind the link between copper and angiogenesis: established mechanisms and an overview on the role of vascular copper transport systems. J. Vasc. Res. 52 (3), 172–196. 10.1159/000438485 26484858

[B41] Vener C. Banzi R. Ambrogi F. Ferrero A. Saglio G. Pravettoni G. (2020). First-line imatinib vs second- and third-generation TKIs for chronic-phase CML: a systematic review and meta-analysis. Blood Adv. 4 (12), 2723–2735. 10.1182/BLOODADVANCES.2019001329 32559295 PMC7322957

[B42] Wang J. Zhao H. Xu Z. Cheng X. (2020). Zinc dysregulation in cancers and its potential as a therapeutic target. Cancer Biol. Med. 17 (3), 612–625. 10.20892/J.ISSN.2095-3941.2020.0106 32944394 PMC7476080

[B43] Wang S. Maxwell C. A. Akella N. M. (2021). Diet as a potential moderator for genome stability and immune response in pediatric leukemia. Cancers 13 (3), 413. 10.3390/CANCERS13030413 33499176 PMC7865408

[B44] Ying H. Zhang Y. (2019). Systems biology of selenium and complex disease. Biol. Trace Elem. Res. 192 (1), 38–50. 10.1007/s12011-019-01781-9 31236815

